# Palps across the tree – the neuronal innervation and development of sensory head appendages in Annelida

**DOI:** 10.3389/fnins.2023.1310225

**Published:** 2024-01-04

**Authors:** Paul Kalke, Samira S. Linder, Patrick Beckers, Conrad Helm

**Affiliations:** ^1^Helm Lab, Johann-Friedrich-Blumenbach-Institute, Animal Evolution and Biodiversity, University of Göttingen, Göttingen, Germany; ^2^Institute of Evolutionary Biology and Zooecology, Evolutionary Biology and Ecology, University of Bonn, Bonn, Germany

**Keywords:** Polychaeta, Magelonidae, Amphinomidae, Spionidae, rostraria, ontogeny, larvae

## Abstract

Polychaetes inhabit a wide variety of habitats and show a great morphological diversity. In this context, a key morphological structure for adapting to their individual lifestyles and ecological niches are the prominent head appendages. In the last years more and more studies focused on the mainly sensory annelid head appendages – namely the antennae, palps, buccal lips and cirri – to unravel the evolutionary origin and phylogeny of Annelida. Unfortunately, comparable data for most of the polychaete families are lacking so far, especially when it comes to features of the larval anterior nervous system and the related innervation and potential homology of these head appendages. In this study, we therefore use an integrative morphological approach including immunohistochemistry and confocal laser scanning microscopy in combination with histological serial sections and 3D-visualizations. With special focus on the palp-like appendages, our data provides a closer look into the development of the larval anterior nervous system and the related sensory structures of three polychaete families representing major groups of the annelid tree of life. Hence, we investigate members of the palaeoannelid Magelonidae as well as basally-branching Amphinomidae, and the pleistoannelid Spionidae forming a taxon deeply nested within Sedentaria. Our comparative data of larval and adult neuronal features support the homology of feeding-palps across the annelid tree. Furthermore, our observations show that larval palps gradually transform into the adult ones while keeping a very similar neuronal innervation pattern. Solely for Amphinomidae a loss of larval palps during ontogenesis has to be assumed. Therefore, our investigations uncover important and so far unknown details in terms of structural homology across Annelida and provide important results necessary for our understanding of annelid evolution.

## 1 Introduction

Marine polychaetes exhibit a remarkable diversity in terms of their morphological adaptations and ecological niches.

**Figure 1:**
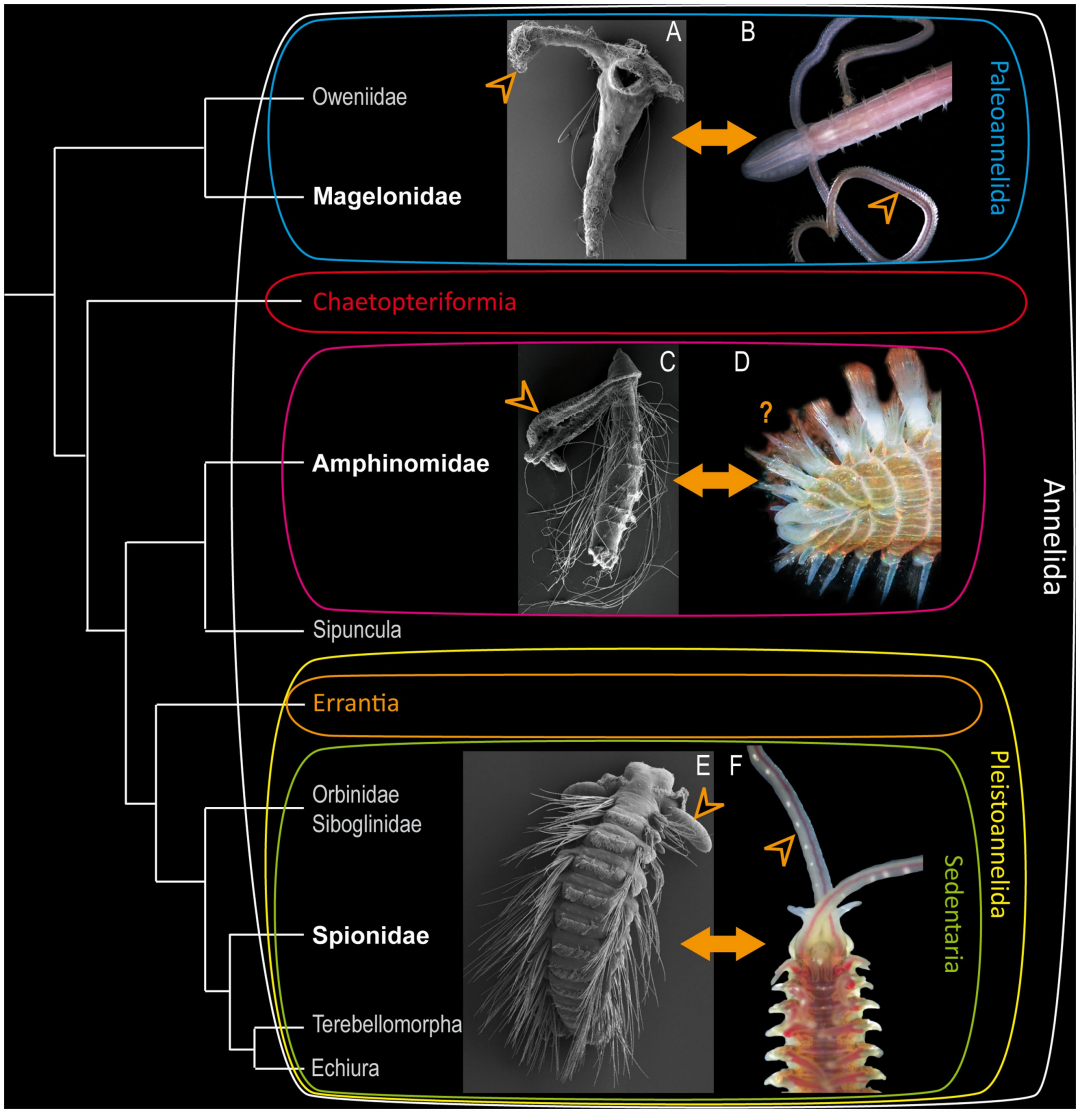
Modified annelid phylogeny based on Helm et al. (2018). **(A)** Larva of *Magelona mirabilis.* **(C)** rostraria larva of *Paramphinome jeffreysii* and **(E)** late larva of *Malacoceros fuliginosus* added as SEM-micrographs. Adults of *Magelona mirabilis* **(B)**, *Eurythoe complanata* **(D)** (Foto: Oliver Mengedoht/Panzerwelten.de) and *Malacoceros fuliginosus* **(F)** added as light microscopic micrographs. The respective families are highlighted in bold white letters, arrowheads mark the position of the larval and adult palps.

In particular the evolution of polychaete head appendages has fascinated researchers across several decades. Nevertheless, the understanding of these structures remains an ongoing scientific endeavor (Nilsson, 1912; Binard and Jeener, 1928; Orrhage, 1966; Rouse and Fauchald, 1997; Rouse and Pleijel, 2001; Orrhage and Müller, 2005; Purschke et al., 2014; Beckers et al., 2019a,b; Kalke et al., 2021). Whereas various aspects of annelid morphology and evolution have been investigated across the years, our knowledge concerning the evolution and putative homology of head appendages in Annelida is still very limited (Purschke et al., 2016). Head appendages – in particular the so-called palps – play a crucial role in the ecological success and adaptability of marine polychaetes, but their morphology and evolutionary adaptation in different taxa is still scarcely investigated (Rouse and Fauchald, 1997; Rouse and Pleijel, 2001; Beckers et al., 2019a,b; Kalke et al., 2021). Interestingly, palp-bearing Annelida either exhibit slender and elongated feeding-palps or stout sensory palps (Purschke et al., 2016). However, the question of a possible homologous evolutionary origin of the different palp types and between similar morphotypes of palps across different taxa still remains questionable (Purschke et al., 2014; Kalke et al., 2021). In particular, the comparison and developmental transition of larval into adult palp-like structures is hardly understood. In this context and as already proposed by Remane (1952) and reviewed by several authors (see Wagner, 1989; Zachos and Hosfeld, 2006), biological homology can be assessed regardless of shape and function. Accordingly, the morphological equivalence in terms of relative position, particular structure as well as internal connections by, e.g., nerves or blood vessels, and the existence of comparable anatomical features in a last common ancestor of the compared species of interest can be taken into account to propose homology of characters. In comparative morphological investigations, in particular the nervous system and its structure, development and general scaffold are often consulted to formulate homology hypotheses within Annelida and other invertebrate taxa (Schmidt-Rhaesa et al., 2015). In the present investigation we therefore focus on the comparison between larval and adult palp-like appendages in three annelid taxa – Magelonidae, Amphinomidae, and Spionidae. These particular families are chosen based on their phylogenetic position and the presence of elongated palp-like head appendages in at least some of the larval stages (see Figure 1). Moreover, at least Magelonidae and Spionidae exhibit elongated palp-like appendages in the adult stage as well.

Accordingly, the basally-branching Magelonidae use their slender palps for an uptake of detritus and organic debris (Jones, 1968; Weigert et al., 2014; Helm et al., 2018). Adult Magelonidae are exceptionally flexible in switching from deposit-feeding (Jones, 1968; Jumars et al., 2015), over subsurface feeding (Jumars et al., 2015) and suspension feeding (Hartmann-Schröder et al., 1971; Wolff, 1973), to carnivorous feeding by catching different larvae (Jones, 1968; Wilson, 1982). As shown previously, adult Magelonidae possess true feeding-palps, which are elongated, possess a food-rim, lack cilia but are papillose, and are neuronally innervated by the dorsal and ventral root of the circumoesophageal connectives (Orrhage, 1966; Purschke et al., 2016; Beckers et al., 2019a). Comparable adult feeding-palps of other taxa exhibiting a similar innervation pattern are also described for, e.g., the basally-branching Oweniidae (Beckers et al., 2019a,b) and Chaetopteriformia (Helm et al., 2022). Therefore, these structures have to be considered as being a plesiomorphic annelid character (Beckers et al., 2019a).

Interestingly, such slender appendages are also present in many taxa of the sedentary Pleistoannelida, such as adult Sabellariidae (Orrhage, 1980; Faroni-Perez et al., 2016), Siboglinidae (Worsaae et al., 2016; Rimskaya-Korsakova et al., 2018), Terebellidae (Orrhage, 2001; Kalke et al., 2021), Spionidae (Orrhage, 1966; Orrhage and Müller, 2005), and Cirratuliformia (Purschke et al., 2016). Nevertheless, our knowledge concerning the larval head appendages is still very scarce.

Many polychaete larvae are planktotrophic (Rouse, 1999, 2006) and thus spend their developmental time in the water column. Many of them develop elongated larval palp-like appendages that are used to capture diatoms, flagellates or other larvae (Wilson, 1936, 1982; Jones, 1968). Although distantly related taxa such as Magelonidae and Spionidae use their multifunctional elongated larval head appendages for feeding throughout various life stages, other annelid taxa bear slender palp-like appendages solely during larval development and lack them in adulthood. One example for such a condition are the pleistoannelid sister group Amphinomidae. In this clade, a transition from planktotrophic larvae (Rouse, 1999, 2006) to omnivorous scavenging or carnivores adults (Fauchald and Jumars, 1979; Jumars et al., 2015) can be observed. This drastic change of ecological adaptations goes along with morphological differences, e.g., in the arrangement of head appendages, the development of sufficient parapodia and hollow, calcified chaetae (Müller et al., 2021). Rostraria larvae – the unique amphinomid larval type - use their elongated palp-like head appendages for ciliary band feeding in a downstream manner (Rouse, 1999, 2006), similar to the larvae of Magelonidae and Spionidae (Wilson, 1982; Dauer, 1983). As adults, most Amphinomidae have two lateral and one median antennae and - as claimed by several authors - two short “palps” located dorsally of the so-called buccal lips (Orrhage, 1990; Orrhage and Müller, 2005). Due to this obvious transformation of head appendages, amphinomids seem to be of particular interest when it comes to the evolutionary transition between the different palp types within Annelida.

By understanding the fate of larval palp-like head appendages and potential transitions in Magelonidae, Amphinomidae, and Spionidae, we herein try to elucidate crucial ontogenetic aspects of these important sensory and feeding structures. By exploring the morphological features and neuronal innervation of palps within and among these families, we aim to shed light on the evolutionary trajectories and developmental changes of these important key structures of annelid evolution.

Our study employs an integrative morphological approach that combines various microscopic techniques such as immunohistochemistry, confocal laser scanning microscopy and Azan-stained histological section stacks, and subsequent 3D-visualization. These methods allow us to visualize and analyze the morphological characteristics of larval and adult palp-like appendages in great detail. For a better understanding and comparison of the main findings, we supply 3D-schemes for the anterior larval neuronal scaffold of all three families.

By doing so we seek to understand the developmental changes and variations of palp types during the transition from larvae to adults, which in addition might give a hint toward the evolutionary origin of the sensory palps found in Errantia. Furthermore, our data will help to answer the fundamental question concerning the biological homology of different types of palp-like appendages and other sensory structures throughout Annelida.

## 2 Materials and methods

### 2.1 Specimen collection

Adult specimens of *Magelona mirabilis* (Johnston, 1865) and *Malacoceros fuliginosus* (Claparede, 1868) were collected in Morgat (Brittany, France) in 2015. Specimens were maintained with the collected sediment in an aquarium with a 12 h:12 h light regime and 3.5% salinity at 17°C at the Michael Sars Centre (Bergen, Norway). Adults were fed a mixture of yeast and ground fish food. After artificial fertilization in filtered seawater (FSW) the developmental stages were reared at 18°C in glass bowls containing 0.5 L FSW. The culture was not aerated, set under strict diurnal rhythm (12:12 – light: dark), and fed with a mix of unicellular algae (*Isochrysis, Nannochloropsis*). Water was changed at least every 2 days. For more details on culturing and artificial fertilization for *Magelona mirabilis* we refer to Beckers et al. (2019a). For details about the culture of *Malacoceros fuliginosus* please see Kumar (2019) and Kumar et al. (2020).

Rostraria larvae of *Paramphinome jeffreysii* (McIntosh, 1868) were collected via a plankton net near the Espegrend marine biological field station (Raunefjord, Bergen, Norway) in 2017. Adult specimens of *Eurythoe complanata* (Pallas, 1766) were collected from the private aquarium of Peter Lesny, Bonn (COI barcode sequence available at the NCBI GenBank, Accession number: KY630466).

### 2.2 Immunohistochemistry

Anatomical details of larvae of *Magelona mirabilis*, *Paramphinome jeffreysii* and *Malacoceros fuliginosus* were investigated using standard immunohistochemical staining protocols. Hence, a minimum of 10 specimens of each species and stage were relaxed in 7% MgCl_2_ and subsequently fixed in 4% paraformaldehyde (PFA) in 1x phosphate buffered saline with Tween (PTW = 1x PBS: 0.05 M PBS / 0.3 M NaCl / 0.3% TritonX. Fixation was performed at room temperature (RT) for 2 h. After fixing, the specimens were washed and stored in PTW containing 0,005% NaN_3_ until usage at 4°C. For antibody staining, specimens were rinsed 3 × 10 min in PTW and incubated in 10 μg proteinase K/ml PTW. Early larvae of *Malacoceros fuliginosus* (10 dpf) and *Magelona mirabilis* (9 dpf) were incubated for 2 min. Rostraria larvae of *Paramphiome jeffreysii* and late larvae of *M. fuliginosus* (60 dpf) were incubated in proteinase-K for 8 min. To stop the proteinase-K reaction, samples were rinsed twice in glycine (2 mg glycine/ml PTW), and washed 3 × 5 min in PTW. Afterwards, the specimens were re-fixed using 4% PFA in PTW containing 0.6/0.3% TritonX for 20 min at RT. Subsequently, the samples were rinsed 2 × 5 min in PTW as well as 2 × 5 min in THT (0.1 M TrisCl, 0.3 TritonX, pH 8,5), and blocked with 5% goat serum (Sigma-Aldrich, Steinheim, 25 μL goat serum in 500 μL THT) for 2 h. Subsequently, specimens were incubated with the primary antibodies against α-tubulin (Anti-acetyl α -tubulin, clone 6-11B-1, Merck, Darmstadt, 2 μL tubulin in 500 μL incl. 5% goat serum) and serotonin (5-HT-serotonin), ImmunoStar Inc., Husk, USA, 1 μL in 500 μL incl. 5% goat serum) in THT for 48–72 h at 4°C. Afterwards, samples were rinsed 2 × 10 min in 1 M NaCl (to deactivate the primary antibody) in THT and washed 5 × 30 min in THT. Subsequently, the samples were incubated in the secondary antibodies goat-anti-mouse 633 (Alexa Fluor® 633 goat-anti-mouse IgG (H + L), Thermo Fisher Scientific Inc., Waltham, USA, 1 μL in 500 μL inlc. 5% goat serum) and goat-anti-rabbit 488 (Alexa Fluor® 488 goat-anti-rabbit IgG (H + L), Thermo Fisher Scientific Inc., Waltham, USA, 1 μL in 500 μL incl. 5% goat serum) in THT for 48–72 h at 4°C. After staining, specimens were rinsed 5 × 30 min in THT and 2 × 5 min in PTW. Additionally, samples were incubated in DAPI (DAPI, Thermo Fisher Scientific Inc., Waltham, USA, 5 μL in PTW) in PTW (we observed better results for DAPI-stainings in PTW) overnight at 4°C. Subsequently, the specimens were dehydrated in an ascending isopropanol series, cleared using Murray’s clear (benzyl alcohol & benzyl benzoate, 1:2) and embedded between two cover slips using DPX mounting medium (Merck, Darmstadt, Germany). The specimens were analyzed with the confocal laser scanning microscopes Leica TCS SP5 and SP8 (Leica Microsystems, Wetzlar, Germany). The confocal image stacks were processed with Leica AS AF v2.3.5 (Leica Microsystems) and Imaris × 64 9.5.0 (Bitplane AG, Zurich, Switzerland).

### 2.3 Azan staining, histological sectioning and 3D-reconstruction

Semi-thin sections and AZAN-staining of adult *Eurythoe complanata* were performed as described previously (Beckers et al., 2013). Accordingly, specimens were relaxed in 7% MgCl2 and then fixed in Bouin’s fluid for 12 h, dehydrated in an ethanol series and incubated in methylbenzoate and butanol. Afterwards, the samples were pre-incubated in Histoplast (Thermo Scientific, Dreieich, Germany) and embedded in Paraplast (McCormick Scientific, Richmond, USA). 5 μm thick sections were made using a Reichert-Jung Autocut 2050 microtome (Leica, Wetzlar, Germany). The sections were transferred to albumen-glycerin coated glass slides. Afterwards, sections were stained with Carmalaun, differentiated with sodium phosphotungstate (5%), washed in distilled water, stained in aniline blue orange G and subsequently embedded with Malinol (Waldeck, Münster, Germany). In Azan staining, the neuropil of the nervous system stain gray, the nuclei of cell somata stain red, the extracellular matrix stain blue, and the musculature stain orange (Beckers et al., 2013). Each section was digitalized at 40x magnification using a slide scanner [Olympus dotslide (2.2 Olympus, Hamburg)] and aligned using IMOD (Kremer et al., 1996) and imodalign.^1^ For the 3D-visualization, we used a digital workflow using solely open source software like ImageJ, MeshLab and Blender (Kalke and Helm, 2022). For the 3D-schemes, we used curve tools in Blender (for a better understanding see: https://www.youtube.com/watch?v=Ve9h7-E8EuM).

## 3 Results

**Figure 2:**
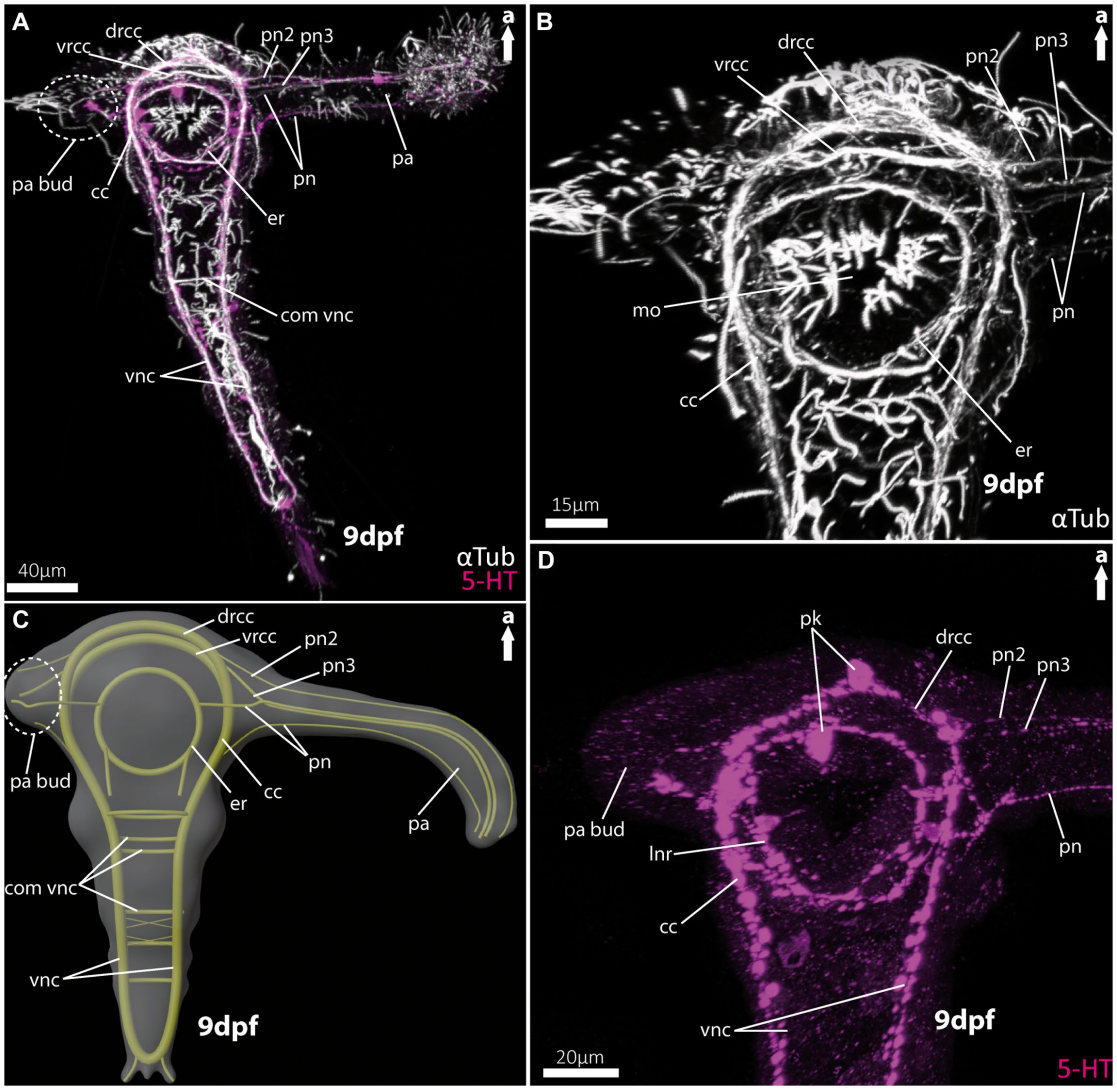
Nervous system of larvae (9 dpf) of the magelonid *Magelona mirabilis*. **(A)** Ventral view of the overall morphology of the nervous system of magelonid larvae stained against acetylated α-tubulin and 5HT-serotonin. The dotted circle marks the growing second palp bud, captured using cLSM micrograph. **(B)** Magnification of the anterior tubulinergic nervous system in ventral view. **(C)** 3D-scheme of the nervous system of the larvae of *Magelona mirabilis* in dorsal view. The dotted circle marks the growing second palp bud. **(D)** Magnification of the serotonergic nervous system in ventral view. cc, circumoesophageal connectives; com vnc, commissure of vnc; drcc, dorsal root of cc; er, oesophageal ring; mo, mouth opening; pa, palp; pa bud, palp bud; pn, palp nerves; pn2, palp nerve of drcc; pn3, palp nerve of vrcc; pk, serotonin-lir perikarya; vnc, ventral nerve cord; vrcc, ventral root of cc.

**Figure 3:**
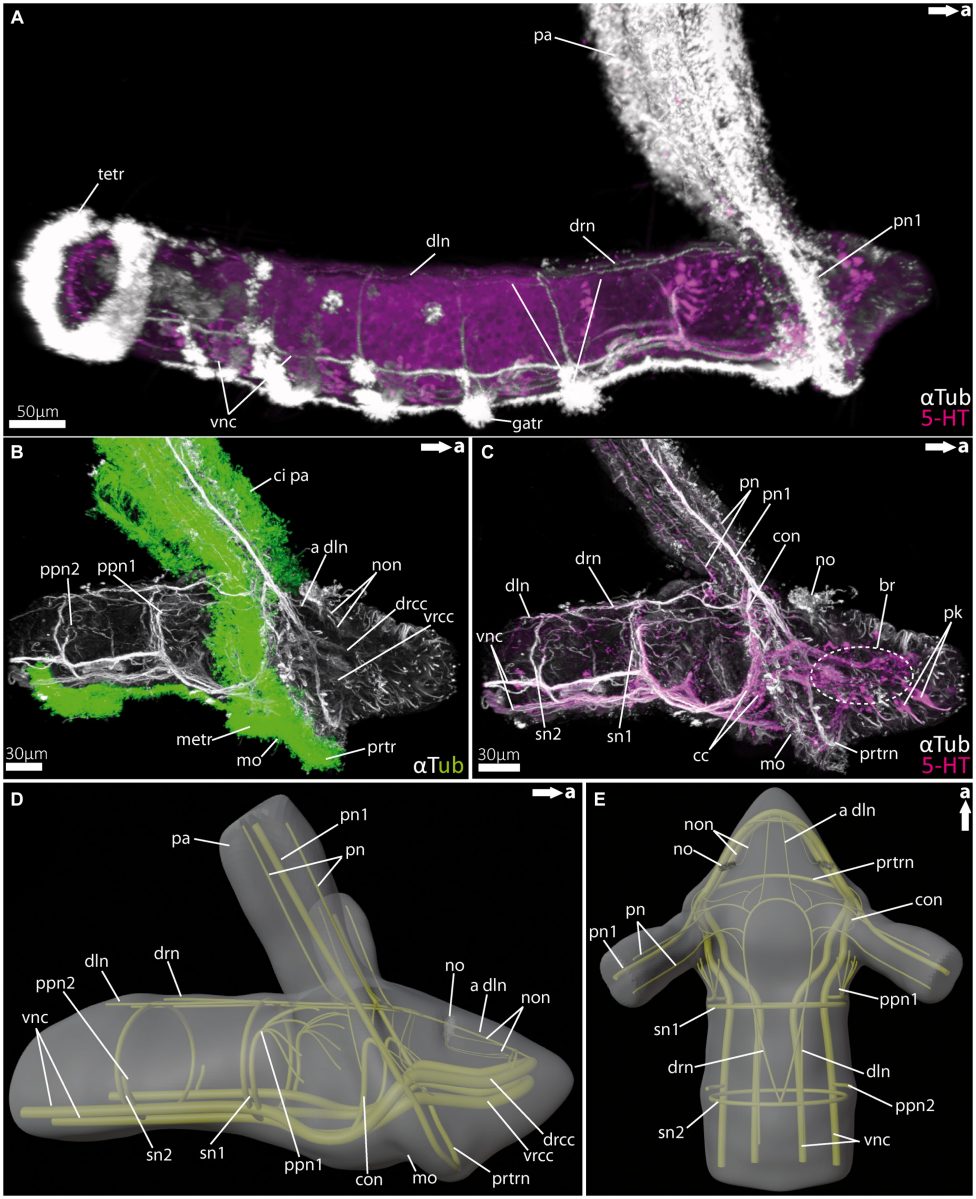
Nervous system of rostraria larvae of the amphinomid *Paramphinome jeffreysii*. **(A)** Overall morphology of the nervous system and ciliation of the rostraria of *Paramphinome jeffreysii* stained against acetylated α-tubulin and 5HT-serotonin, showing only one third of the strong ciliated feeding-palps. **(B)** Magnification of the anterior nervous system stained against acetylated α-tubulin, highlighting cilia in green, cLSM micrograph. **(C)** Same magnification of the anterior nervous system as in **(B)** stained against acetylated α-tubulin and 5HT-serotonin, omitting the cilia of the elongated feeding-palps and the ciliary bands. The dotted circle marks the position of the brain, captured using cLSM micrograph. **(D,E)** 3D-scheme of the anterior nervous system of the rostraria. **(D)** In lateral and **(E)** in dorsal view. a dln, anterior dorsal longitudinal neurite bundle; br, brain; cc, circumoesophageal connectives; ci pa, cilia of palps; con, drcc + pn1 connecting neurite bundle; dln, dorsal longitudinal neurite bundle; drcc, dorsal root of cc; drn, dorsal ring nerve; gatr, gastrotroch; metr, metatroch; mo, mouth opening; no, nuchal organ; non, nuchal organ nerves; pa, palp; pn, palp nerves; pn1, main palp nerve interconnected with drcc and vrcc; ppn1, parapodial neurite bundle of 1st chaetiger; ppn2, parapodial neurite bundle of 2nd chaetiger; prtr, prototroch; prtrn, prototroch nerve; pk, serotonin-lir perikarya; sn, segmental neurite bundles; sn1, segmental neurite bundle of 1st chaetiger; sn2, segmental neurite bundle of 2nd chaetiger; tetr, telotroch; vnc, ventral nerve cord; vrcc, ventral root of cc.

**Figure 4:**
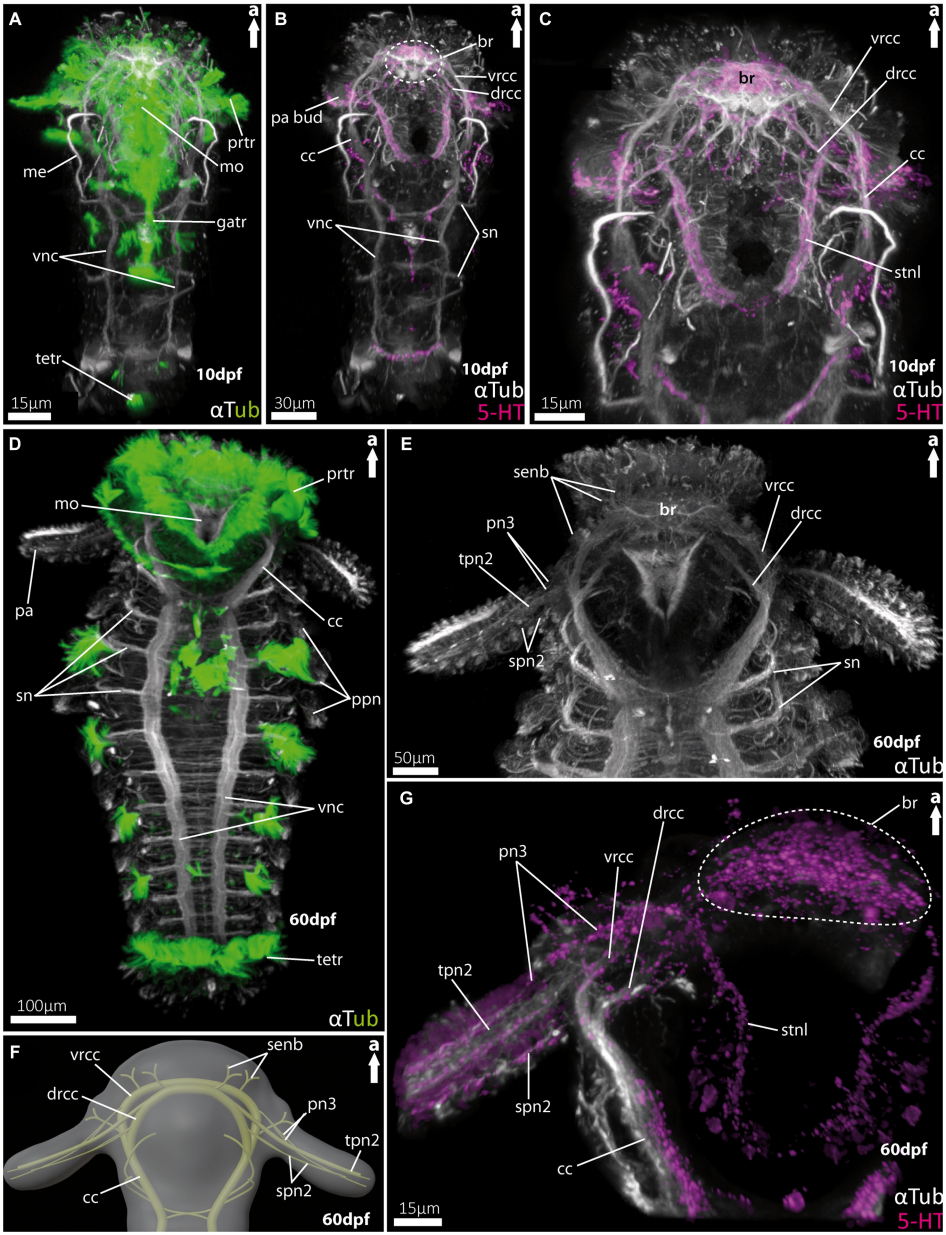
Nervous system of larvae of the spionid *Malacoceros fuliginosus*. **(A)** Overall morphology of the tubulinergic nervous system of the metatrochophore (10 dpf) in ventral view, with ciliary bands highlighted in green, captured using cLSM micrograph. **(B)** Overall morphology of the nervous system of a metatrochophore (10 dpf) stained against acetylated α-tubulin and 5HT-serotonin in ventral view. The dotted circle marks the position of the brain, and early palp buds start to develop directly under the prototroch, cilia omitted captured using cLSM micrograph. **(C)** Magnification of the anterior nervous system of the metatrochophore shown in **(B)**, with the dorsal and ventral roots of the circumoesophageal connectives well displayed. **(D)** Overall morphology of the tubulinergic nervous system and external ciliation (highlighted in green) of a pre-metamorphic specimen (60 dpf) of *M. fuliginosus* close to its metamorphosis in ventral view. **(E)** Magnification of the anterior tubulinergic nervous system with a focus on the brain and innervation of the feeding-palps in ventral view. **(F)** 3D-scheme of the anterior nervous system of a pre-metamorphic larvae of the same age in dorsal view. **(G)** Magnification of the anterior nervous system stained against acetylated α-tubulin and 5HT-serotonin of pre-metamorphic *M. fuliginosus* in dorsal view. br, brain; cc, circumoesophageal connectives; drcc, dorsal root of cc; gatr, gastrotroch; me, metanephridia; metr, metatroch; mo, mouth opening; pa, palp; pa bud, palp bud; tpn2, thick palp nerve of drcc; spn2, slender palp nerve of drcc; pn3, palp nerve of vrcc; ppn, parapodial neurite bundles; prtr, prototroch; senb, sensorial neurite bundles; sn, segmental neurite bundles; stnl, stomatogastric nerve loop; tetr, telotroch; vnc, ventral nerve cord; vrcc, ventral root of cc.

**Figure 5:**
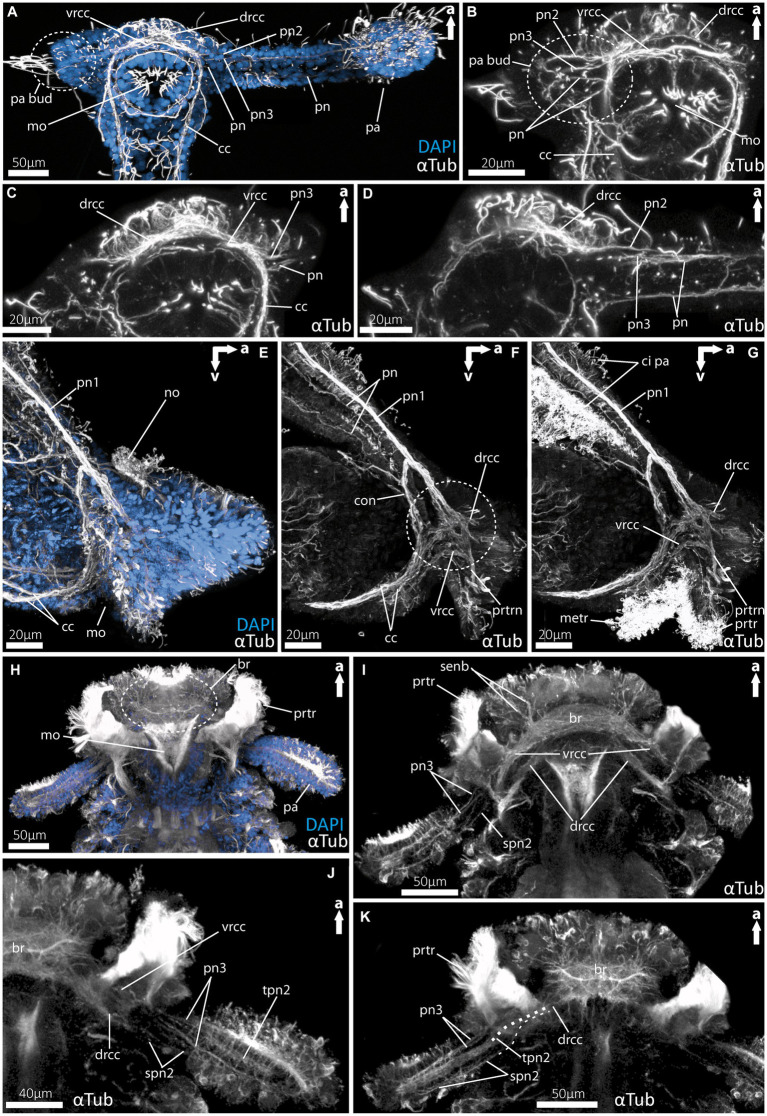
Details of the palp innervation patterns of 9dpf (days post fertilization) larvae of *Magelona mirabilis* (Magelonidae), rostraria larvae of *Paramphinome jeffreysii* (Amphinomidae) and 60 dpf larva of *Malacoceros fuliginosus* (Spionidae). **(A)** Overview of the anterior tubulinergic nervous system of *Magelona mirabilis* counterstained with DAPI (nuclei). **(B)** Digital sections through the palp bud of *M. mirabilis* showing the innervation of the latter by the *drcc, vrcc, and cc*. **(C)** Digital section through the proximal part of the larval palp of *M. mirabilis* showing the origin of *pn3* from the ventral root and the anterior *pn* from the *cc.* **(D)** Digital section posterior to **(C)** showing the origin of *pn2* from the dorsal root of the *cc*. **(E)** Overview of the anterior tubulinergic nervous system of rostraria larva of *P. jeffreysii* counterstained with DAPI. **(F)** Digital section from lateral through the right palp-like appendage, dashed circle marks the position of the strong interconnection of the *pn1* with the dorsal and ventral root of the *cc*. **(G)** Same section with ciliation showing the prototroch and metatroch surrounding the mouth opening and cilia of the palp-like appendages. **(H)** Overview of the anterior tubulinergic nervous system of pre-metamorphic larva of *Malacoceros fuliginosus* counterstained with DAPI. **(I)** Digital section through the anterior end showing the origin of the palp nerves 3 from the ventral root of the *cc.* **(J)** Digital section through the anterior end posterior to **(I)** showing all palp nerves and the origin of the two *spn2* from the dorsal root of the *cc*. **(K)** Digital section through the anterior end posterior to **(J)** showing all palp nerves and the origin of *tpn2* and *spn2* from the dorsal root of the *cc.* Dashed line visualize the origin from the d*rcc* of *spn2* and *tpn2*. br, brain; cc, circumoesophageal connectives; ci pa, cilia of palps; con, drcc + pn1 connecting neurite bundle; drcc, dorsal root of cc; metr, metatroch; mo, mouth opening; pa, palp; no, nuchal organ; pa bud, palp bud; pn, palp nerves; pn1, main palp nerve interconnected with drcc and vrcc; pn2, palp nerve of drcc; pn3, palp nerve of vrcc; prtr, prototroch; prtrn, prototroch nerve; senb, sensorial neurite bundles; senb, sensorial neurite bundles; spn2, slender palp nerve of drcc; tpn2, thick palp nerve of drcc; vrcc, ventral root of cc.

**Figure 6:**
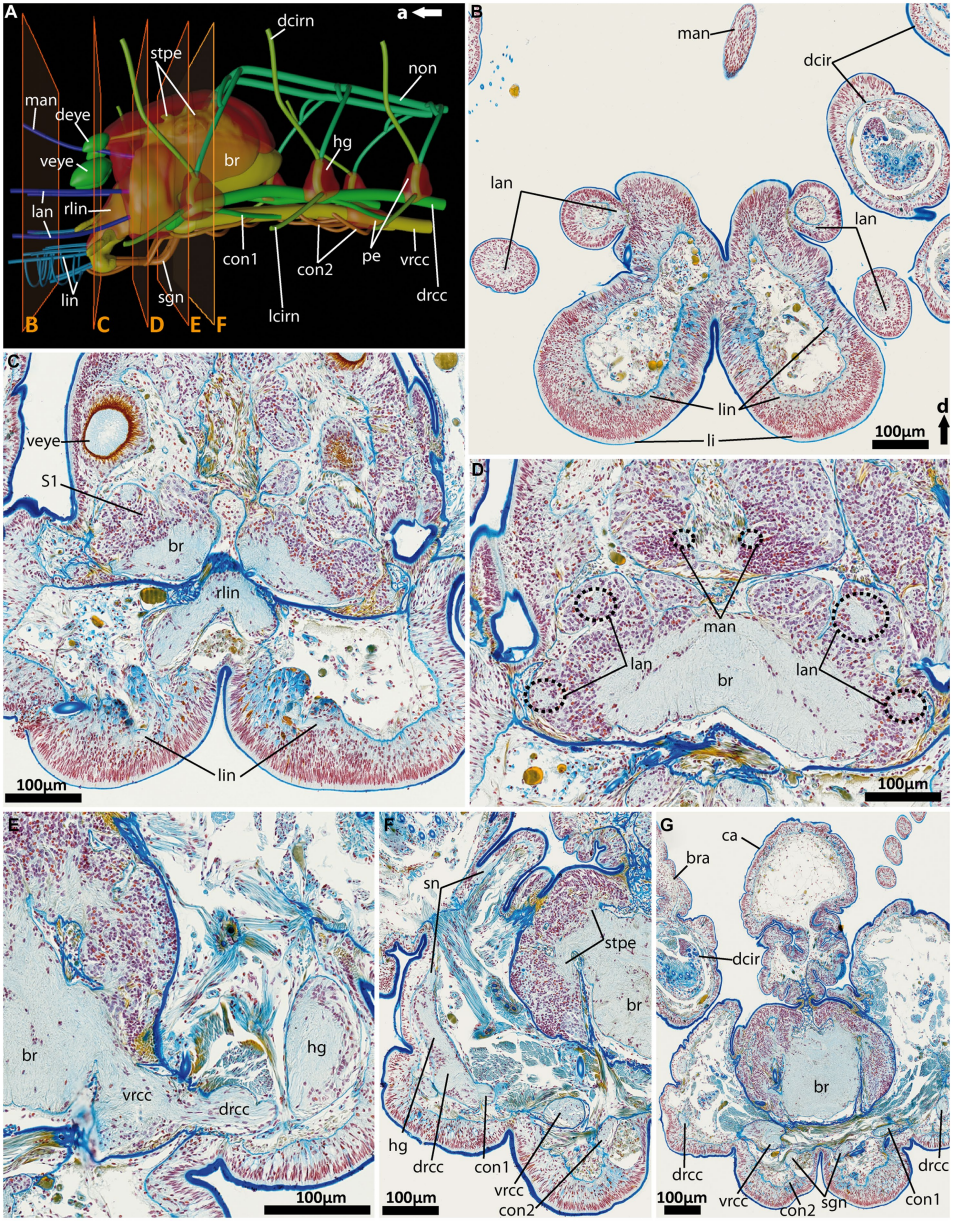
General anatomy with focus on the nervous system of the anterior end of adult *Eurythoe complanata*. **(A)** 3D-scheme of the anterior nervous system of adult *E. complanata* in lateral view. The frames show the area of Azan-stained 5 μm paraffin-sections **(B–F)**, light microscopic images. **(B)** Cross-section through the paired lips and all five antennae, see **(A)**. **(C)** Cross-section through the root of the main lip neurite bundles (rlin) and the anterior region of the complex brain, see **(A)**. **(D)** Cross-section through the brain region all five antennae nerves originate. **(E)** Cross-section through area where *vrcc* and *drcc* enter the brain. **(F)** Cross-section through the first hemi-ganglion, splitting off. **(G)** Cross-section through the caruncle and the brain slightly posterior to the area where the nuchal organ nerve (*non*) leaves the brain. br, brain; bra, branchiae; ca, caruncle; con1, neurite bundle connecting drcc and vrcc; con2, neurite bundle connecting vrcc and sgn; dcir, dorsal cirrus; dcirn, lcirn, nerves of dorsal and lateral cirrus; deye, dorsal eye; drcc, dorsal root of cc; hg, hemi-ganglion; lan, nerve of lateral antenna; li, lips; lin, lip neurite bundle; man, nerve of median antenna; non, nuchal organ nerve; pe, perikarya; rlin, root of lip neurite bundle; sn, segmental neurite bundle; sgn, stomatogastric neurite bundles; stpe, stalk of perikaria cluster; veye, ventral eye; vrcc, ventral root of cc; S1, type 1 neuron refer to Beckers and Tilic (2021).

**Figure 7:**
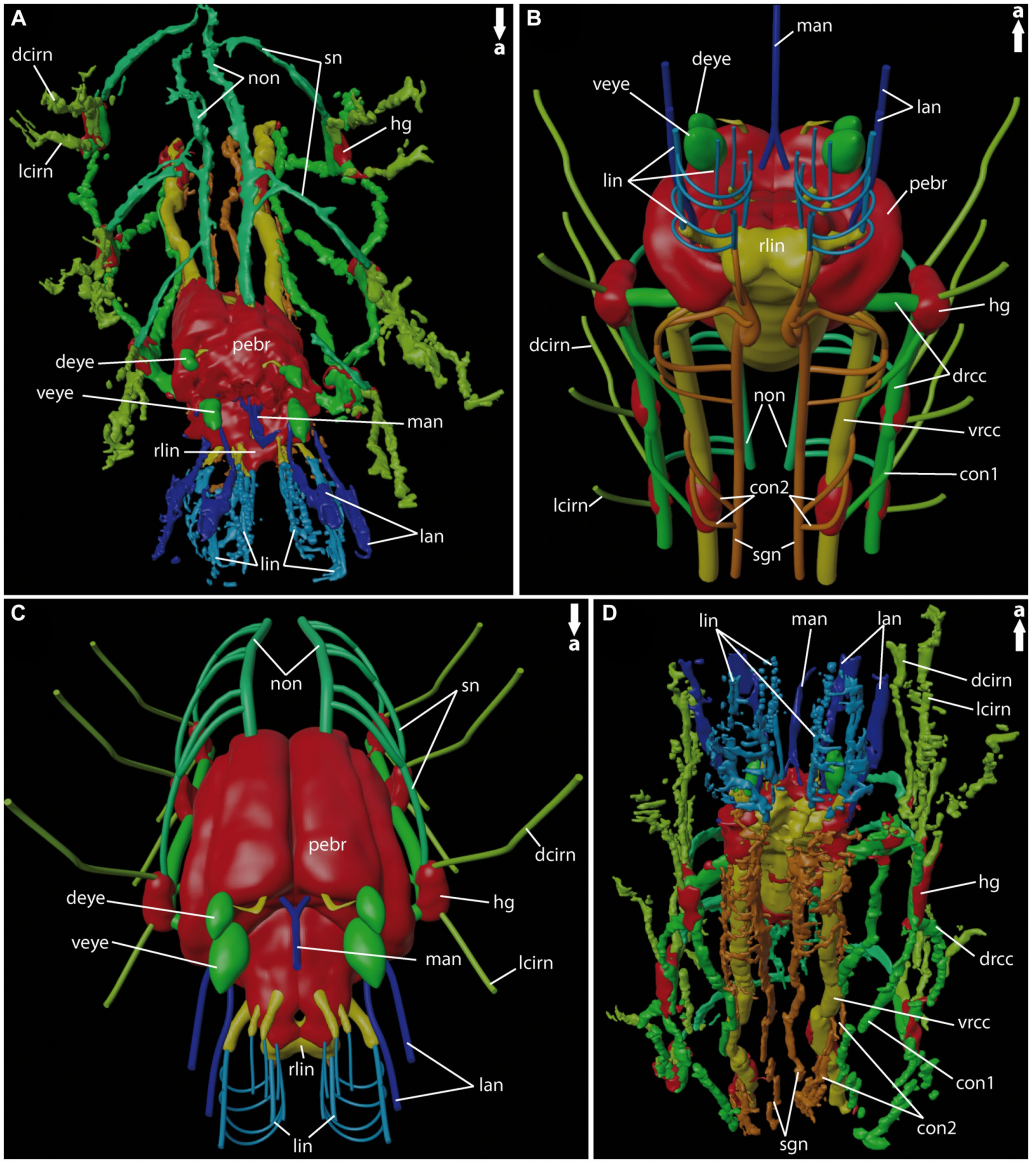
General anatomy of the anterior nervous system of adult *Eurythoe complanata.* **(A)** 3D-reconstruction based on Azan-stained paraffin-sections in antero-dorsal view. **(B)** 3D-scheme of adult *E. complanata* in antero-ventral view. **(C)** 3D-scheme of adult *E. complanata* in antero-dorsal view. **(D)** 3D-reconstruction based on Azan-stained paraffin-sections in ventral view. con1, neurite bundle connecting drcc and vrcc; con2, neurite bundle connecting vrcc and sgn; dcirn, lcirn, nerves of dorsal and lateral cirrus; deye, dorsal eye; drcc, dorsal root of cc; hg, hemi-ganglion; lan, lateral antenna; li, lips; lin, lip neurite bundle; man, median antenna; non, nuchal organ nerve; pebr, perikarya of brain; rlin, root of lip neurite bundle; sn, segmental neurite bundle; sgn, stomatogastric neurite bundles; veye, ventral eye; vrcc, ventral root of cc.

For the investigation of larval and adult anterior morphology, we used 9 days-post-fertilisation (9 dpf) larvae of the magelonid *Magelona mirabilis* (Jonston, 1865) (Figure 2), planktotrophic rostraria larvae of the amphinomid *Paramphinome jeffreysii* (McIntosh, 1868) (Figure 3), and 10 dpf as well as 60 dpf larvae of the spionid *Malacoceros fuliginosus* (Claparède, 1868) (Figure 4). Details of neuronal innervation patterns of the larval palp-like head appendages are summarized in Figure 5. Adult *Eurythoe complanata* (Pallas, 1766) were used for Azan-histological sections (Figure 6) and subsequent 3D-reconstruction (Figure 7). For a better understanding of morphological features, we provide videos of the 3D-reconstruction as well as the 3D-visualization in the Supplementary files.

For general terms and annotations of the nervous system characters, we refer to Richter et al. (2010), for the anterior nervous system we used the annotations of Schmidbaur et al. (2020). For the description of adult *E. complanata* we refer to the nomenclature used in Beckers and Tilic (2021).

### 3.1 Central nervous system and head appendages in larval *Magelona mirabilis* (Magelonidae)

As already known from the literature, the early larvae of *M. mirabilis* show one prominent palp-like appendage (Figures 2A,C, 5A), whereas the other one is still in development and only appears as a palp-bud in 9-dpf larvae (Figures 2A,C, 5A,B). Both structures are randomly ciliated and do not exhibit a constant and uniform ciliation pattern known for ciliary bands in other annelid larvae. The anterior central nervous system consists of the ventral (*vrcc*) and dorsal root (*drcc*) of the circumoesophageal connectives (*cc*) and unify in posterior direction to the *cc* (Figures 2A–C, 5A–D). The transition of the circumoesophageal connectives (*cc*) into the ventral nerve cord (*vnc*) is fluent (Figures 2A–D). The *vnc* already shows three commissures (Figures 2A,C) and terminates posteriorly by generating two pygidial nerves.

The well-developed palp-like appendage is innervated by at least four neurite bundles. The two most prominent ones (*pn2* and *pn3*) originate from the *vrcc* and *drcc* (Figures 2A–D, 5C,D). The two more slender palp neurite bundles originate from the esophageal ring (*er*) - which surrounds the mouth opening (Figures 2A–C, 5C) – and from the *cc* more posteriorly (Figures 2A–D, 5D). Serotonin-like immunoreactivity (serotonin-lir) is observed in most structures of the anterior central nervous system, excluding the *vrcc*. The esophageal ring (*er*) shows a strong serotonergic signal too, crowned by one of two prominent perikarya. The second prominent serotonergic perikaryon is located anterior-most on the *drcc*. Although the *vrcc* shows no serotonergic signal, all neurite bundles innervating the palp-like appendage do (Figure 2D).

### 3.2 Central nervous system and head appendages in rostraria larvae of *Paramphinome jeffreysii* (Amphinomidae)

Rostraria larvae exhibit two characteristic and strongly ciliated palp-like appendages (Figures 3A,B), which extend over the length of its entire body (not shown in full length). Similar to many polychaete larvae, rostraria larvae also show a posterior telotroch, a ventral gastrotroch and a more anteriorly arranged meta- and prototroch (Figures 3A,B). The prototroch – which is situated anterior to the mouth opening- and the metatroch in a more posterior position, surround the larval anterior end ventrally and give rise to the ciliated food-rim of the palps and mouth region (Figures 3B, 5G). The larval brain possesses a prominent ventral (*vrcc*) and dorsal root (*drcc*) of the circumoesophageal connectives (Figures 3B–D). The latter form distinct neurite bundles in posterior direction, termed by doing so as circumoesophageal connectives (*cc*) and ventral nerve cord (*vnc*) (Figures 3A–E). In a regular manner circular, segmental neurite bundles interconnect the neurite bundles of the *vnc* and the dorsal longitudinal neurite bundle (*dln*) (Figures 3A,C–E). Directly anterior of the serial neurite bundles, paired parapodial nerves are situated (Figures 3B,D,E). The larval nuchal organs are located anterior of the palp-like larval appendages and are innervated by two slender neurites bundles originating from the dorsal *drcc* (Figures 3C–E).

The distinct larval palp-like structures are innervated by at least three palp nerves (Figures 3A,C–E, 5E,F). The most prominent palp nerve (*pn1*) represents the continuation of the prototroch nerve (*prtrn*), which runs along the upper lip (Figures 3C,D, 5F,G). *Pn1* continues in dorsal direction crossing the *vrcc* and *drcc* (Figures 3B–D, 5F,G). At this point, slender neurite bundles from both roots fuse with *pn1* (Figures 3B–D, 5F). In addition, one prominent neurite bundle (*con*) originates from the *drcc* and fuses with *pn1*, too (Figures 3C,D, 5F). The two slender palp nerves (*pn*) run (like *pn1*) along the palp but split after entering the head region dorsally and fuse next to *pn1* and *con* with the dorsal ring nerve (*drn*) (Figures 3C–E).

The *drn* is located dorsally (between the palps) and is highly interconnected, appearing as a kind of intersection between both bilateral symmetrical sides of the animal (Figure 3E). Thus, it is connected anteriorly and laterally with the *drcc.* Laterally, there are further interconnections with *con* and *pn1*, and posteriorly with the *dln* and segmental nerves.

### 3.3 Central nervous system and head appendages in larvae of *Malacoceros fuliginosus* (Spionidae)

For *Malacoceros fuliginosus* we refer to the quite early metatrochophore at 10 dpf (days-post- fertilization) as well as the late pre-metamorphic larva at 60 dpf. The early metatrochophore represents typical larval features such as a distinct prototroch, gastro- and telotroch. Furthermore, the first pair of metanephridia is developed at this stage (Figure 4A). The palp buds are visible posterior to the prototroch by anti-serotonin staining (Figure 4B; note that the cilia are removed in this image). The anterior central nervous system is well developed. It exhibits a distinct brain region consisting of the ventral (*vrcc*) and dorsal root (*drcc*) of the circumoesophageal connectives (*cc*), which fuse posteriorly to the latter (Figures 4B,C). The transition from the *cc* into the ventral nerve cord (*vnc*) is fluent and first slender commissures and segmental nerves appear (Figures 4A,B).

In pre-metamorphic larvae of *M. fuliginosus*, the larval ciliary bands are still well developed (Figures 4D, 5H). The *vnc* shows two strong and distinct neurite bundles with α-tubulin-lir bearing ~70 slender commissures and at least 12 segmental nerves (Figure 4D). The transition from the *vnc* to the *cc* is distinct and in addition marked by the first two anterior-most segmental neurite bundles (Figures 4E,F). In anterior direction, the *cc* splits into the *vrcc* and the *drcc* (Figures 4E–G, 5I). Dorsally, each root of the *cc*, most likely, sends sensorial nerves into the anterior most part of the anterior end (Figures 4E,F, 5I).

The pre-metamorphic palps are innervated by at least five neurite bundles (Figures 4E–G, 5I–K). Two slender nerves originate anteriorly from the *vrcc*. The *drcc* gives rise to one thick nerve and two slender ones, which – in contrast to the thick one – emerge slightly more posterior from the *drcc* (Figures 4E,F, 5I–K). The serotonin-lir shows the most-prominent signal in the entire brain region. The palp nerves (*pn3*) originate from the *vrcc,* and the stomatogastric nerve loop is also innervated by the *vrcc* in close vicinity (Figure 4G).

### 3.4 Central nervous system and head appendages of adult *Eurythoe complanata* (Amphinomidae)

Adult *E. complanata* possess five antennal appendages – four lateral and one median one (Figures 6A, 7A–D). Distally, all antennae exhibit circular nerves, which split into two neurite bundles and fuse, in the lateral antennae, again immediately after entering the anterior end of *E. complanata* (Figures 6A, 7A–D). Posterior to the origin of the root of the lip neurite bundles (*rlin*), all six antennal nerves fuse with the dorsal main brain mass (Figures 6B–D, 7A–D). The paired lobed lips are innervated by at least six longitudinal neurite bundles, which all originate from different locations of the *rlin* and are interconnected by numerous semi-circular neurite bundles (Figures 6A–C, 7A–D). Anterior of the main brain mass, *E. complanata* exhibits two ventral big pigmented eye spots and two smaller dorsal ones, which are innervated by neurite bundles originating from the antero-dorsal brain (Figures 6A,B, 7A–C).

The brain itself is complex and highly compressed. Therefore, the assignment of a distinct ventral (*vrcc*) and dorsal root (*drcc*) of the circumoesophageal connectives in the brain or their respective tracts is intricate based on our dataset. Posterior to the fusion of the *rlin*, the *vrcc* and *drcc* proceed into the central neuropil, which increases in size and appeares tapered in posterior progression (Figures 6A, 7A–D). At the level where *vrcc* and *drcc* enter the brain, several rows of dorso- and dorso-lateral stalk-like neurite bundles originate from two sack-like clusters of perikarya (Figures 6A,F, 7C). These two rows of bilateral clusters are closely associated with each other and proceed to the posterior end of the brain neuropil (Figures 6A, 7A–C).

In comparison with other annelids, the arrangement of the ventral nerve cord (*vnc*) is quite unique in *Eurythoe*. As already described for the rostraria of *P. jeffreysii* the *vrcc* and *drcc* proceed longitudinally in distinct and separated neurite bundles, although entering the brain in close vicinity (Figures 6A,E, 7B). Both neurite bundles are regularly interconnected by the *con1*, which runs from the hemi-ganglion of the *drcc* in posterior direction and fuse with the *vrcc* (Figures 6F,G, 7B,D). The segmentally arranged hemi-ganglia give rise to the lateral (*lcirn*) and dorsal cirral nerves (*dcirn*) of the parapodia (Figures 6A,G, 7A–D). From the *dcirn* the nerves of the segmental branchiae split off. The hemi-ganglia of the at least first three segments are interconnected to the paired nuchal organ nerves (*non*) by the hemi-circular segmental nerves (*sn*) (Figures 6F, 7A,C). The huge *non* proceed dorsally along the anterior–posterior axis toward groups of undefined cell clusters (not shown) giving rise to several slender neurite bundles running into the caruncle (Figures 6A,G, 7A–C). The origin of the *non* is posteriorly, in close proximity to the dorsal-most row of stalk-like neurite bundles connecting the two rows of perikarya with the dorsal neuropil of each brain side. In addition to the *vrcc* and the *drcc,* another paired neurite bundle, located medially (*sgn*), can be observed (Figures 6A,G, 7B,D). This stomatogastric neurite bundles innervate the alimentary canal more posteriorly (not shown) and are interconnected with the *vrcc* via the *con2* multiple times (Figures 6A,F,G, 7B,D). Anteriorly, the *sgn* fuse with the paired root of the lip nerves (*rlin*), together with at least one longitudinal lip nerve from the opposing side (Figures 6A, 7B,D).

For a better understanding of the described structures, please check the Supplementary files S1, S2.

## 4 Discussion

### 4.1 The larval anterior nervous system and sensory head appendages in Magelonidae, Amphinomidae, and Spionidae

The larval anterior nervous systems and head appendages of Magelonidae, Amphinomidae, and Spionidae exhibit several common neuronal characteristics that can be observed in various taxa. One notable feature is the presence of prominent and well-defined lateral circumoesophageal connectives, consisting of a dorsal and ventral root. These connectives fuse at the anterior end and run into the brain. The brain region of the spionid *Malacoceros fuliginosus* shows a quite complex and structured neuropil compared to *Magelona mirabilis* and *Paramphinome jeffreysii*. However, all observed larval stages share certain developmental characteristics, such as the existence of several larval ciliary bands and the occurrence of a simple but well-developed ventral nerve cord (*vnc*).

Nevertheless, one interesting ontogenetic aspect is the onset of larval palp development, which drastically varies among the taxa investigated. Notably, the larval feeding-palps appear quite late in the development of *Malacoceros* when compared to *Magelona*, where the latter develop ontogenetically early (before 5 dpf), but not simultaneously (see also Beckers et al., 2019a). Hence, solely one larval palp is present in early stages, whereas the second one develops quite late in the planktonic phase (see also Wilson, 1982). For *P. jeffreysii* it remains difficult to estimate palp appearance due to the capture method and the longevity of rostraria larvae (Rouse, 2006; Barroso et al., 2010). However, the external anatomy of the rostraria and the neuronal innervation patterns observed herein, suggest a similar developmental stage as shown for pre-metamorphic palp-bearing *Malacoceros* larvae.

Despite these variations, the examined late larval stages possess well-developed feeding-palps. Furthermore, these feeding-palps appear elongated and well-defined by external morphological features such as a prominent food-rim (Purschke et al., 2014). Additionally, the neuronal innervation of all investigated larval palps is highly comparable and consistent, with innervating neurite bundles originating from the dorsal (*drcc*) and ventral root (*vrcc*) of the circumoesophageal connectives (Orrhage and Müller, 2005).

### 4.2 Comparison of the adult anterior nervous system and head appendages of Magelonidae, Amphinomidae, and Spionidae

The head appendages of adult Magelonidae and Spionidae have been extensively studied, and due to their prominent appearance and external similarity, both taxa were often considered as being closely related in morphological analyses (Rouse and Fauchald, 1997). Although finally assigned to distantly related annelid clades based on phylogenomic analyses (Weigert and Bleidorn, 2016), the elongated feeding-palps of both taxa fulfill all the morphological criteria to be termed as such (see Orrhage, 1966; Beckers et al., 2019a). Accordingly, the palps are elongated and have a food-rim or are “grooved” (Rouse and Fauchald, 1997; Rouse and Pleijel, 2001; Struck, 2011; Purschke et al., 2014). Furthermore, the slender palps in both taxa are innervated by distinct neurite bundles originating from the *drcc* and *vrcc* and their respective commissures (Orrhage and Müller, 2005) - criteria which are also fulfilled for the head appendages of other basally-branching annelid groups, such as Owenidae (Beckers et al., 2019a,b) and Chaetopteridae (Orrhage, 1966; Helm et al., 2022). Therefore, this characters and in particular the neuronal innervation has to be suggested as being a plesiomorphic character state for Annelida (Beckers et al., 2019a). Furthermore, these features are also known for a variety of sedentary pleistoannelids (Orrhage and Müller, 2005; Purschke et al., 2016; Kalke et al., 2021). As a consequence, the adult feeding-palps containing a food-rim have to be considered as being homologous throughout Annelida, because all criteria defining biological homology can be applied. Accordingly, the relative position and structure as well as internal neuronal innervation patterns of all adult feeding-palps investigated so far appear highly similar and are traceable along the entire annelid tree. For annelid taxa with a different type of palp-like appendages, such as Errantia or the previously investigated in Terebelliformia (Kalke et al., 2021), the picture is not that homogeneous at the first glance.

Another exception represents the anterior nervous system and the respective head appendages of *Eurythoe complanata* (Amphinomidae), which exhibit unique characteristics when compared to other polychaetes. In adult amphinomids, the dorsal (*drcc*) and ventral root (*vrcc*) of the circumoesophageal connectives (*cc*) form distinct neurite bundles, similar to the conditions observed in the rostraria of *Paramphinome jeffreysii*. Additionally, the *drcc* was referred to as the “lateral nerve” (Müller et al., 2003; Helm et al., 2018) or “longitudinal podial nerve” (Orrhage, 1990), but due to the conserved separation of *vrcc* and *drcc* in larvae and adult specimens a change in naming had to be proposed. Our data suggest that the *drcc* contributes to the brain formation, a fact which is also supported by previous data (Orrhage, 1990). Other studies dealing with the regeneration of the anterior amphinomid nervous system show that the “lateral nerve” originates from the ventral nerve cord of the amputee and takes part in the formation of the regenerating *drcc* of the brain (Müller et al., 2003; Helm et al., 2018). Nevertheless, in *E. complanata* the *drcc* and *vrcc* never fuse to a common *cc*, despite encircling the oddly posterior located mouth opening and oesophagus (see Figure 1D).

Exceptional is also the arrangement of the stomatogastric neurite bundles (*sgn*) - a third pair of longitudinal neurite bundles, that proceed parallel between the strands of the *vrcc*. These bundles innervate the stomatogastric tract further posterior (own observation, Orrhage, 1990) and are connected to the *vrcc* multiple times. In the anterior direction, the stomatogastric neurite bundles fuse with the roots of the lip nerves. As mentioned previously, annelid stomatogastric nerves always originate from the *vrcc*, which seems to be true for amphinomids as well (Kalke et al., 2021).

The buccal lips – structures situated ventrally at the anterior tip of the amphinomid head - are innervated from longitudinal nerves solely emanating from distinct lip nerve roots, which are part of the *vrcc*. Although, we cannot rule out the contribution of nerves emanating from the *drcc*, our data seems to resemble observations made for Eunicidae and *Hyalinoecia tubicola* (Onuphidae) as well (Zanol, 2010; Kuhl et al., 2022). Nevertheless, further data are needed to support a homology of these structures in the different taxa.

When it comes to the antennal appendages, the neurite bundles of the five antennae of *E. complanata* originate from the main dorsal fibril mass. As already described (Orrhage, 1995; Orrhage and Müller, 2005), the nerves of all antennae originate solely from the dorsal root of the *cc*. For *Eurythoe complanata*, the origin of all antennal neurite bundles from the same brain region in direct vicinity to the dorsal-most commissure of the *drcc* can be considered as an additional indicator. However, in our data we do not observe any contribution of neurite bundles emanating from the *vrcc*. Therefore, our findings cannot confirm previous investigations, that annotate the ventral-most lateral antennal appendages as being palps innervated by both roots of the circumoesophageal connectives (Orrhage, 1990).

Antennae in general are only described for amphinomids and pleistoannelids, and seem to be evolved in the last common ancestor of both (and Sipuncula) (Purschke et al., 2016). The presence of five antenna-like appendages appears to be a rare characteristic, and besides amphinomids only certain errant Eunicida (Zanol, 2010) show a similar condition. Notably, recent investigations dealing with the neuronal innervation patterns of head appendages of Eunicida support the presence of a maximum of three antennae in Polychaeta (Kuhl et al., 2022) – a condition that was previously emphasized (Orrhage and Müller, 2005; Purschke et al., 2014). Consequently and supported by our presented data, amphinomids seem to be the only polychaetes examined so far that exhibit five antennae.

Accordingly, we disagree with the use of the term “palps” for the description of the lateral-most head appendages, as assumed for Amphinomidae by previous authors (Orrhage, 1990). Instead, our data supports the ontogenetic reduction of adult palps in amphinomids, a point that will be discussed in the next paragraph.

### 4.3 The fate of larval head appendages and homology of annelid feeding-palps

The larval neuroanatomy and neuronal innervation pattern of head appendages as herein observed for Magelonidae (and Spionidae) is highly similar to the larval and/ or adult conditions exhibited by basally-branching annelid taxa, such as Oweniidae (Beckers et al., 2019a,b), Chaetopteridae (Helm et al., 2022), and Amphinomidae (present study). In other Pleistoannelida, this neuronal pattern is often retained in adult or late juvenile specimens (Orrhage and Müller, 2005; Purschke et al., 2016).

In the particular case of Magelonidae, previous studies suggested that larvae shed their larval palps during metamorphosis and re-grow them as adults (Wilson, 1982). Additionally, Wilson mentioned that “very late larval stages taken in plankton hauls often lack larval tentacles these may have been shed as a result of crowded, sometimes silty, conditions in the tow-net bucket” – a fact which would explain the observation of short, growing palps in adults post metamorphosis. Based on our observations as well as the identical neuronal innervation pattern of in larval and adult palps, a gradual development of larval toward adult feeding-palps has to be assumed. Therefore, a loss of larval palps during metamorphosis in Magelonidae seems implausible and might have been caused by destructive sampling methods during earlier investigations.

A similar condition with the larval feeding-palps sharing exactly the same innervation pattern as described for adult individuals is supported for *Malacoceros fuliginosus* (Orrhage, 1966). All criteria of biological homology – namely the relative position, particular structure and development as well as internal connections such as neurite bundles and the putative existence of comparable anatomical features in a last common ancestor – can be applied (Remane, 1952). Consequently, the larval as well as the adult palps of Magelonidae and Spionidae have to be considered as being homologous to the feeding-palps of other annelid taxa - both throughout the annelid tree and the entire ontogeny.

In Amphinomidae, a different picture can be observed. The enigmatic rostraria larvae of *Paramphinome jeffreysii* exhibit true elongated feeding-palps, which exhibit a feeding-palp-like neuronal innervation pattern as observed in other annelid taxa. Accordingly, the larval feeding-palps of Amphinomidae are homologous to other annelid feeding-palps as well.

Nevertheless, in terms of the neuroanatomy described for the anterior end of adult amphinomids (in this case *E. complanata*), no structure with a neuronal innervation pattern of the larval feeding-palps, can be observed. As a consequence, a reduction or loss of the larval feeding-palps during metamorphosis in Amphinomidae has to be assumed – a finding that changes our understanding in terms of amphinomid morphology as well as the putative homology assumptions that were made in the past. Accordingly, adult amphinomid specimens do not possess head appendages that can be homologized with feeding-palps (based on their neuronal innervation pattern and development). Nevertheless, the transitional reduction of the respective structures in this annelid family helps to understand the evolutionary adaptive changes in terms of palp morphology throughout annelid taxa.

Although our comparative investigations of feeding-palp-like appendages in different families and developmental stages clearly support a homology of feeding-palps throughout the annelid tree, other questions concerning the evolution of palp-like appendages in Annelida remain unanswered. In particular, our knowledge concerning the evolution and putative homology of the stout sensorial palps of Errantia (Pleistoannelida) is still scarce and needs further investigations.

## 5 Conclusion

Our comparative study elucidates that the comparison of larval versus adult features facilitates our understanding of evolutionary transitions of morphological key structures in Annelida and helps to draw conclusions concerning developmental changes due to ecological adaptations. Accordingly, our integrative analyses clearly support a homology of adult and larval feeding palps throughout members of the distantly related Palaeoannelida, Amphinomidae and Sedentaria (Pleistoannelida). Additionally, our observations highlight the adaptive reduction of these feeding-palps within amphinomid development and elucidate the immense diversity when it comes to annelid evolution. Our data nicely illustrate, how drastic the changes during metamorphosis can turn out, if the ecological adaptations of larvae and adults differ. Nevertheless, more comparative studies are needed to fill the knowledge gap of many other annelid taxa, in particular for the ontogenetic and evolutionary changes that took place within the branch leading toward Errantia.

## Data availability statement

The original contributions presented in the study are included in the article/Supplementary material, further inquiries can be directed to the corresponding authors.

## Ethics statement

Ethical approval was not required for the study involving animals in accordance with the local legislation and institutional requirements because not applicable by the use of polychaetes.

## Author contributions

PK: Conceptualization, Data curation, Formal analysis, Investigation, Methodology, Visualization, Writing – original draft, Writing – review & editing. SL: Investigation, Writing – review & editing. PB: Data curation, Investigation, Methodology, Resources, Validation, Writing – review & editing. CH: Conceptualization, Investigation, Project administration, Resources, Supervision, Validation, Writing – review & editing.

## Footnotes

^1^ www.q-terra.de/biowelt/3drekon/guides/imod_first_aid.pdf

## Supplementary material

Supplementary File 1 Video-file of the 3D-reconstruction based on Azan-stained histological section series of the anterior end of adult *Eurythoe complanata*, shown in Figures 7A,D.

Supplementary File 2 Video-file of the 3D-scheme based on the 3D-reconstruction (see S2) of the anterior end of adult *Eurythoe complanata*, shown in Figures 7B,C.
